# Stabilization of Hypoxia-Inducible Factor Promotes Antimicrobial Activity of Human Macrophages Against *Mycobacterium tuberculosis*

**DOI:** 10.3389/fimmu.2021.678354

**Published:** 2021-06-02

**Authors:** Sebastian F. Zenk, Sebastian Hauck, Daniel Mayer, Mark Grieshober, Steffen Stenger

**Affiliations:** Institute of Medical Microbiology and Infection Control, University Hospital Ulm, Ulm, Germany

**Keywords:** tuberculosis, hypoxia, HIF, human, macrophages, Molidustat

## Abstract

Hypoxia-inducible factor (HIF) is a key oxygen sensor that controls gene expression patterns to adapt cellular metabolism to hypoxia. Pharmacological inhibition of prolyl-hydroxylases stabilizes HIFs and mimics hypoxia, leading to increased expression of more than 300 genes. Whether the genetic program initialized by HIFs affects immune responses against microbial pathogens, is not well studied. Recently we showed that hypoxia enhances antimicrobial activity against *Mycobacterium tuberculosis* (*Mtb*) in human macrophages. The objective of this study was to evaluate whether the oxygen sensor HIF is involved in hypoxia-mediated antimycobacterial activity. Treatment of *Mtb*-infected macrophages with the prolyl-hydroxylase inhibitor Molidustat reduced the release of TNFα and IL-10, two key cytokines involved in the immune response in tuberculosis. Molidustat also interferes with the p38 MAP kinase pathway. HIF-stabilization by Molidustat also induced the upregulation of the Vitamin D receptor and human β defensin 2, which define an antimicrobial effector pathway in human macrophages. Consequently, these immunological effects resulted in reduced proliferation of virulent *Mtb* in human macrophages. Therefore, HIFs may be attractive new candidates for host-directed therapies against infectious diseases caused by intracellular bacteria, including tuberculosis.

## Introduction

Tuberculosis is an airborne infectious disease caused by *Mycobacterium tuberculosis* (*Mtb*), which primarily affects human lungs. Active tuberculosis is treated with a combination of isoniazid, rifampin, pyrazinamide and ethambutol for at least 6 months to achieve clearance of the pathogen and prevent the selection of drug resistant mutants (1). Drug resistance against *Mtb* was already described in 1948 when the very first human TB therapy trial using Streptomycin was conducted (2). In 2014 (3) *Mtb* isolated from approximately 1.9 million patients were isoniazid mono-resistant (13.3%) and multidrug resistant (MDR, 5.3%). 9.7% of individuals with MDR-TB had extensively drug-resistant (XDR) tuberculosis, which was reported by 105 countries (WHO, 2020). Infection with drug resistant *Mtb* requires longer and more-toxic treatment and is only moderately effective. Hence there is an urgent need for the development of novel strategies to treat tuberculosis (4). Modern concepts include host-targeted therapies to promote immune responses without toxicity and development of drug resistance.

HIFs are not only sensors for cellular hypoxia, but also control key functions of immune cells required for protection against microbial pathogens (5, 6). Though several HIF isoforms exist, HIF-1α is the most prominent and detected nearly in all innate immune populations (7). Under normoxia (20% O_2_) HIF-1α is rapidly degraded by prolyl-hydroxylases, von Hippel-Lindau tumor suppressor protein and the proteasome (8). Hypoxia (pO_2_ ≤1%) deactivates prolyl hydroxylases and consequently HIF-1α is stabilized and translocated into the nucleus. Here, the transcription of multiple target genes responsible for angiogenesis (e.g. vascular endothelial growth factor), cellular proliferation (e.g. erythropoietin), glucose metabolism (e.g. glucose transporters) as well as inflammation (e.g. inflammatory cytokines) are induced (9, 10).

Recently, others and we demonstrated that hypoxia is beneficial for the control of *Mtb* in macrophages obtained from humans and non-human primates (11, 12). Furthermore, pharmacological induction of hypoxia by VEGF-signaling in a *Mycobacterium marinum* zebrafish model reduced bacterial growth *in vivo* (13). There is evidence that HIF-1α plays an important role in innate immune responses directed against a wide variety of pathogens including group A and B streptococci, *Staphylococcus aureus*, *Salmonella typhimurium*, *Pseudomonas aeruginosa* and Mycobacteria (14). The myeloid HIF-response influences metabolism (cellular ATP pool), production of granule proteases (neutrophil elastase, cathepsin G), expression of antimicrobial peptides (cathelicidin), inducible nitric oxide and cytokines (TNFα, IL-1, IL-4, IL-6, IL-12) (7, 15–17). Recently we demonstrated that hypoxia upregulates an antimicrobial effector pathway mediated by the vitamin D receptor (VDR) and human β defensin 2 (hBD2) (12).

Proly hydroxylase inhibitors can be applied to stabilize HIFs in normoxic atmosphere and induce downstream antimicrobial effector functions. The HIF-stabilizers L-Mimosine and AKB-4924 showed therapeutic benefit in mouse models of *Staphylococcus aureus* skin infection (18, 19), and dimethyloxaloylglycine supported host defense in a *Mycobacterium marinum* zebrafish model (17). Currently several prolyl hydroxylase-inhibitors (FG-2216, Roxadustat, Daprodust, Molidustat and AKB-6548) are under evaluation in clinical trials or already approved for the treatment of renal anemia (20–23).

Given the complex downstream events orchestrated by HIF, any pharmacological manipulation of this pathway must consider potential harmful effects for the host, including susceptibility to microbial pathogens. Here, we investigated whether HIF-stabilization by the prolyl-hydroxylase inhibitor Molidustat modulates the immune response of human macrophages against the major human pathogen *Mtb*. Our results demonstrate a differential effect of Molidustat on the release of cytokines (reduction) and the VitD-mediated antimicrobial effector pathway (induction) ultimately resulting in reduced intracellular growth of virulent *Mtb*. These findings suggest that HIF-stabilization promotes the antimicrobial function of human macrophages and this pathway may provide a new target for host-directed therapies against tuberculosis.

## Materials and Methods

### Cell Culture and Reagents

Primary human cells were cultured in RPMI 1640 (Life Technologies) supplemented with 2 mM glutamine (Sigma), 10 mM HEPES, 13 mM NaHCO_3_, 100 µg/ml streptomycin, 60 µg/ml penicillin (all from Biochrom) and 5% heat-inactivated human AB serum (Sigma) (complete medium). For THP-1 cells (ATCC^®^ TIB-202™, Institute for Medical Microbiology and Hygiene, Ulm University) heat-inactivated human AB serum was replaced by 20% fetal calf serum ([FCS] Sigma). For experiments involving the virulent laboratory strain *Mtb* H37Rv (ATCC^®^ 27294™, Institute for Medical Microbiology and Hygiene, Ulm University) the medium was modified to optimize phagocytosis (non-heat-inactivated serum) and allow multiplication of the bacteria (no streptomycin). In order to prevent fungal growth 5.6 µg/ml Amphotericin B and 60 µg/ml Penicillin G were added. *Mtb*-extract was used as source for soluble mycobacterial antigens in a final concentration of 10 µg/ml. These antigens were generated by collecting the ultracentrifuged supernatant of mycobacterial cells that were repeatedly sonicated. Afterwards cell wall components and intracellular antigens were extracted (24). Molidustat (BAY 85-3934, Selleckchem) is a HIF-stabilizer (prolyl-hydroxylase inhibitor), which was dissolved in DMSO and serially diluted in PBS.

### Hypoxia Chamber

A hypoxia chamber was tailor-made for the specific requirements of a biological safety level 3 facility (Toepffer Laboratories). The chamber represents a closed system that is accessible from the outside through aerosol tight gloves. Outgoing air is filtered through a high-efficiency particulate air (HEPA Class 14) filter to permit experiments with virulent *Mtb*. Materials and reagents are shuttled into the chamber *via* a sluice, such that the atmosphere remains constant at all times during the experiments. Temperature (37°C), CO_2_ (5%), and humidity were constant, and O_2_ and N_2_ were adjusted according to the experimental requirements. All parameters were monitored by digital sensors.

### Preparation of Macrophages and THP-1 Cells

Peripheral blood mononuclear cells (PBMC) were isolated by density gradient centrifugation of buffy coat preparations from anonymous donors (Institute of Transfusion Medicine, Ulm University). Macrophages were generated from plastic-adherent PBMC cultured in the presence of granulocyte-macrophage colony-stimulating factor (GM-CSF, 10 ng/ml, Miltenyi) for 7 days. Macrophages were stored in liquid nitrogen if required. THP-1 cells (ATCC, TIB-202™) were differentiated to macrophages by treatment with phorbol 12-myristate 13-acetate (10ng/ml) for 18hrs.

### Culture of Mycobacterium Tuberculosis

*Mtb* H37Rv was grown in suspension with gentle rotation in roller bottles containing Middlebrook 7H9 broth (BD Biosciences) supplemented with 1% glycerol (Roth), 0.05% Tween 80 (Sigma), and 10% Middlebrook oleic acid, albumin, dextrose, and catalase enrichment (BD Biosciences). Aliquots from logarithmically growing cultures were frozen in PBS/10% glycerol, and representative vials were thawed and enumerated for viable colony forming units (CFU). *Mtb* were sonicated in a pre-heated (37°C) water bath for 10 min prior to use.

### Quantification of Extracellular and Intracellular Mycobacterial Growth

To determine the effects of Molidustat on extracellular *Mtb*, bacteria were cultured in 96 well plates in 7H9 broth. Iron supplementation was accomplished by addition of filtered (0.2 µm pores, Millipore) iron (II) sulfate heptahydrate (FeSO_4_ * 7 H_2_O, Sigma). *Mtb* were then incubated for 5 days. Subsequently extracellular bacteria were harvested by vigorous re-suspension, transferred into screw caps, and sonicated in a preheated (37°C) water bath for 10 min. Aliquots of the sonicate were serially diluted (1:10, 1:100, 1:1000) in cell culture medium without streptomycin. Four dilutions of each sample were plated on 7H11 agar plates (BD Biosciences) and incubated for 14 days before determining the number of CFU. To analyze the effects of Molidustat on intracellular *Mtb*, macrophages were infected with single-cell suspensions of *Mtb* at a multiplicity of infection (MOI) of 10 as bulk infection. After 18 hrs macrophages were washed with PBS to remove extracellular bacteria. Viability of macrophages was verified with the LIVE/DEAD^®^ Kit (Invitrogen) and was approximately 95%. The percentage of infected macrophages was controlled regularly by acid-fast stain and was between 31 ± 17% with 1-10 bacilli per infected cell. Infected macrophages were then equally distributed into the wells of a culture plate before adding the stimuli which secures an equal bacillary burden in all samples at the beginning of the incubation period. After 5 days of culture, the number of viable bacilli was determined by plating cell lysates (0.3% saponin; Sigma) on 7H11 agar plates as described above.

### Viability Assays

Annexin V/propidium iodide staining was performed using the “FITC Annexin V Apoptosis Detection Kit I” from BD Biosciences following the manufacturer’s protocol. Data were analyzed by flow cytometry (FACSCalibur, BD) using FlowJo, version 10.1 (Tree Star Inc., Ashland, Oregon, USA).

### Measurement of Cytokine Concentrations

Supernatants were harvested, and stored at -70°C. Supernatants from *Mtb*-infected cultures were filtered (0.2 µm) and sterility was confirmed by culture of filtered aliquots on 7H11 agar plates. The concentration of tumor necrosis factor α (TNF-α) and interleukin-10 (IL-10) was determined by enzyme linked immunosorbent assay (ELISA, R&D Systems) exactly as suggested by the manufacturer. Sensitivity of the ELISAs was regularly 32 pg/ml.

### Western Blot Analysis

Macrophages (5-10 x 10^6^/ml) were incubated with Molidustat and *Mtb*-extract for 16 ± 2 hrs. Subsequently cells were harvested with ice-cold PBS 1 mM EDTA and centrifuged (4°C, 16.000 g for 20 s). Pellets were lysed using CER I buffer (NE-PER™, Thermo Scientific), protease inhibitor cocktail tablets as well as phosphatase-inhibitor cocktail tablets (both from Roche). For HIF-1α detection proteins were purified directly from CER I buffer lysates containing nuclear and cytoplasmic protein fractions. Deferoxamine (DFO, Sigma) was used in a final concentration of 50 µM in order to prevent Prolylhydroxylase II mediated degradation of HIF-1α by Fe^2+^ chelation. Protein concentrations of lysates were adjusted to equal levels according to BCA Protein Assay (Pierce). Lysates containing 25–100 µg protein were boiled (10 min, 95°C) in Laemmli sample buffer (2 mM SDS (Roth); 0.3% Glycerol (Sigma); 63 mM Tris-HCl (Sigma), pH 6; 0.03% bromophenol blue (Biomol); 0.15% mercaptoethanol (Sigma)) and analyzed by SDS-Page (12%) and western blot. Membranes were probed overnight with HIF-1α mAb mouse (BD Biosciences) (1:1000), respectively. Afterwards donkey anti mouse alkaline phosphatase conjugated antibody (1:10.000, Jackson ImmunoResearch Laboratories) was used as secondary antibody. Proteins were detected by chemiluminescence (CDP Star, Roche) following the manufacturer’s protocol. The same membrane was stripped (0.2 M Glycine (AppliChem GmbH); 3.5 mM SDS (Roth); 0.1% Tween 20 (Sigma), pH 2.2) and re-probed using β-Actin rabbit mAb (Cell Signaling Technology) (1:1000).

Phosphorylated p 38 (pp38) detection was performed with 7 x 10^5^ THP-1 macrophages after 16 ± 2 hrs treatment as indicated. Processing of lysates was performed as described above. Membranes were probed overnight with pp38 rabbit polyclonal Ab (Cell Signaling Technology) (1:1000). Band intensity was measured by using Image J software (Version 1.50i).

### Quantitative RT- PCR

Macrophages (2,5 - 5 x 10^6^/ml) were incubated with Molidustat in presence or absence of *Mtb*-extract for 48 h. Cells were lysed and RNA was prepared using the RNeasy purification kit (Qiagen) according to the manufacturer’s instructions. cDNA was prepared by reverse transcription (Fermentas). LightCycler PCR was performed using SYBR Green PCR Master Mix (Roche). The following primers were used: forward

β-actin, 5’-GGCCACGGGCTGCTTC-3’; reverse β-actin, 5’ GTTGGCGTACAGGTCTTTGC-3’; forward vitamin D receptor (VDR), 5’-AAGGACAACCGACGCCACT-3’; reverse VDR, 5’-ATCATGCCGATGTCCACACA-3’; forward hBD2, 5’ GGTGTTTTTGGTGGTATAGGCG-3’; reverse hBD2, 5’-AGGGCAAAAGACTGGATGACA-3’. The cycles were performed as follows: 1 cycle 95°C, 10 min; 40 cycles: 95°C, 15 s; 60°C, 10 s; 72°C, 20 s. The mRNA levels for VDR and hBD2 were normalized to the amount of β-actin, which was measured simultaneously. A comparative threshold cycle was used to determine gene expression relative to untreated cells cultured at 20% O_2_. Relative expression levels were calculated using the delta-delta CT method (∆∆CT) as described previously (25).

### Statistical Analysis

Experiments were performed 5-11 times as indicated in the respective legends. For each experiment macrophages were derived from independent anonymous blood donors. Results are presented as mean ± standard error of the mean (SEM). Statistical significance was calculated using the Student’s *t* test for paired samples or one tailed Wilcoxon test as indicated. Differences were considered significant if *p ≤* 0.05.

## Results

### Molidustat Is Not Toxic for Primary Human Macrophages

**Figure 1:**
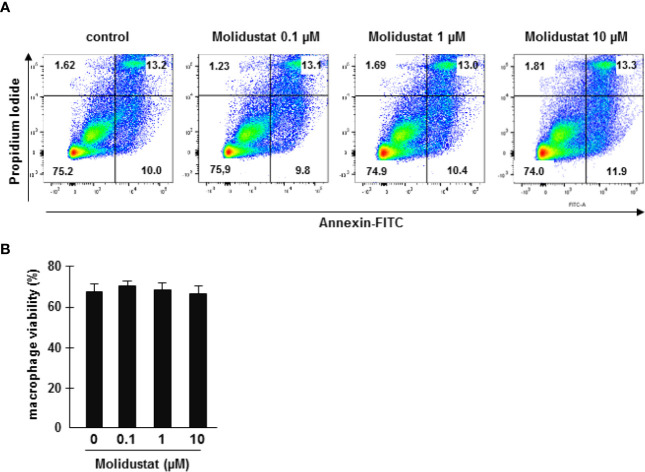
Molidustat is not toxic for primary human macrophages. **(A)** 1x10^6^ macrophages were incubated with medium alone or increasing levels of Molidustat as indicated. After 16 ± 2 hrs incubation apoptotic and necrotic cells were detected by annexin V-FITC and PI-staining. The panel shows a representative result of seven independent donors. **(B)** The diagram gives the average percentage ± SEM of annexin V and PI negative macrophages of seven independent donors. There were no statistical differences as calculated with a Wilcoxon test.

To rule out that stabilization of HIF by the prolyl hydroxylase inhibitor Molidustat affects the viability of macrophages, cells were incubated overnight in the presence of increasing concentrations of Molidustat (0.1 µM to 10 µM). Viability was evaluated by Annexin-FITC/Propidium Iodide staining and analyzed by flow cytometry (**Figure 1A**). Cell viability in general was slightly reduced (70%) due to the purification- and culture conditions. Importantly, Molidustat had no effect on the viability of macrophages as compared to un-treated cells generated from 7 independent donors at all concentrations tested (**Figure 1B**).

### Molidustat Stabilizes HIF-1α in *Mtb*-Treated Macrophages

**Figure 2:**
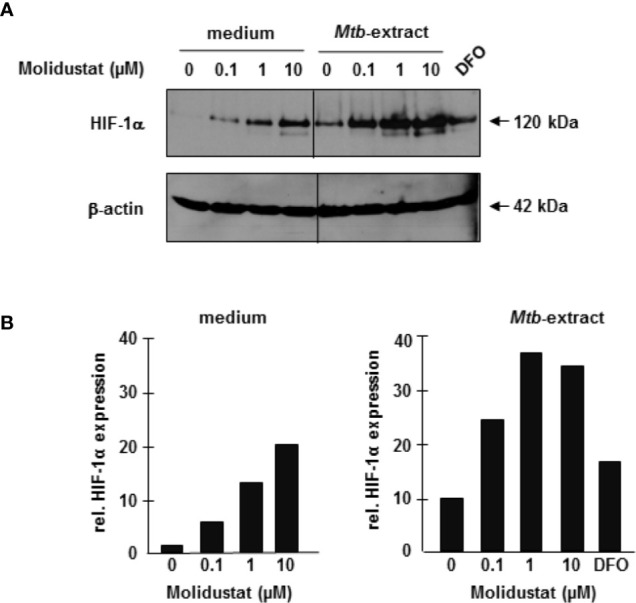
Molidustat stabilizes HIF-1α in *Mtb*-stimulated macrophages 2×10^6^ macrophages were stimulated for 16 ± 2hrs with Molidustat in the absence or presence of *Mtb*-extract (10 µg/ml) or Deferoxamine (DFO, 50 µM). Cell lysates were probed with a HIF-1α antibody (1:1000). **(A)** The upper panel shows a representative result of three independent experiments. The lower panel shows the blots after stripping and detection of β-actin (1:1000). **(B)** The graphs show the relative HIF-1α expression calculated by measuring the integrated densities as compared to un-treated samples (defined as 1).

To ascertain that Molidustat-mediated prolyl-hydroxylase inhibition increases HIF-1α expression in the specific cell population used in our study, macrophages were incubated overnight with 0.1 µM, 1µM and 10 µM Molidustat. HIF-1α expression was determined by Western Blot analysis. HIF-1α expression was increased by Molidustat in a dose dependent manner (**Figures 2A, B**). Since the major objective of this study was to investigate the effect of HIF-1α-stabilization on *Mtb*-mediated immune responses, we next stimulated macrophages with mycobacterial antigens (*Mtb*-extract) in the absence or presence of Molidustat. *Mtb*-extract alone stabilized HIF-1α and the expression was further enhanced in a dose-dependent manner by treatment with Molidustat (**Figures 2A, B**) and reached levels beyond deferoxamine, which prevents prolyl hydroxylase II mediated degradation of HIF-1α by Fe^2+^ chelation and was therefore used as a positive control for HIF-1α stabilization. These results demonstrate that microbial antigens stabilize HIF-1α in human macrophages and this effect is enhanced by the prolyl hydroxylase inhibitor Molidustat.

### HIF-Stabilization Reduces *Mtb*-Mediated Cytokine-Release and Leads to an Interference With p38 MAP Kinase Activation

**Figure 3:**
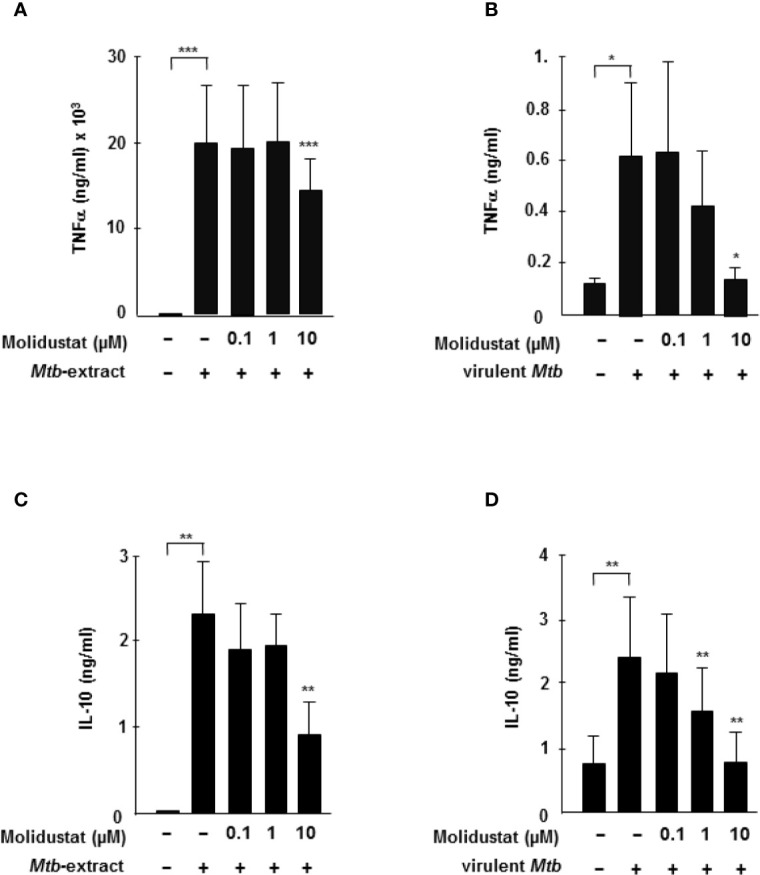
HIF-stabilization reduces *Mtb*-mediated TNFα and IL-10 release **(A, C)** 3 x 10^5^ macrophages were either left untreated or stimulated with *Mtb*-extract (10µg/ml) and incubated with increasing concentrations Molidustat for 16 ± 2 hrs. The release of TNFα or IL-10 in the supernatant was determined by Elisa. The figures present the cytokine release (mean ± SEM) of 10 (TNF-α) and 5 (IL-10) independent donors. **(B, D)** 3 × 10^5^ macrophages were infected with virulent *Mtb* at an MOI of 10. TNFα and IL-10 concentrations in the supernatants were determined by ELISA after 16hrs of infection. The figures present the cytokine release (mean ± SEM) of 7 independent donors. Asterisks indicate statistical significance as calculated by Wilcoxon test. *p ≤ 0.05; **p ≤ 0.01; ***p ≤ 0.001.

Since HIF-1α is stabilized by mycobacterial antigens, we hypothesized that pharmacological manipulation of HIF-expression by Molidustat might affect *Mtb-*mediated macrophage activation. Since cytokine release is an essential function of macrophages in the immune response against intracellular bacteria, we analyzed the effects of HIF-stabilization on the *Mtb*-induced release of the TNFα and IL-10. We selected TNFα and IL-10 because both are key mediators for orchestrating the immune response in human tuberculosis (26). Molidustat alone did not induce the release of TNF-a or IL-10 (not shown). *Mtb*-extract induced high levels of TNFα-release by macrophages (19 ng/ml ± 7 ng/ml) (**Figure 3A**). Treatment with Molidustat (10 µM) reduced *Mtb*-extract-mediated TNFα-release by 26% (n=10). Importantly, Molidustat also inhibited the release of TNFα by macrophages infected with virulent *Mtb* to background levels (**Figure 3B**). Similarly, the *Mtb*-extract induced release of IL-10 was inhibited by Molidustat (61 ± 16%; 7 donors, **Figure 3C**). Furthermore, Molidustat reduced IL-10 secretion induced by viable *Mtb* in a dose-dependent manner and suppressed the cytokine release to background levels at a concentration of 10 µM (7 donors, **Figure 3D**). Therefore, the stabilization of HIF-1α by Molidustat results in the reduced secretion of TNFα and IL-10, supporting our hypothesis that HIF-1α is a potential target for the modulation of innate immune responses in *Mtb*-infection.

**Figure 4:**
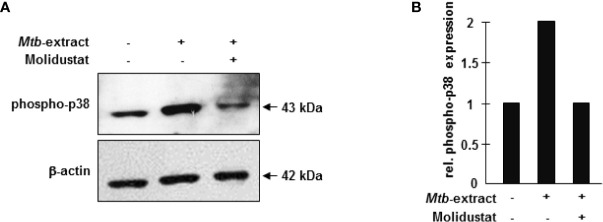
HIF-stabilization interferes with p38 MAP kinase activation. **(A)** 7x10^5^ THP-1 macrophages were either left untreated or stimulated with 10 µg/ml *Mtb*-extract in absence or presence of 10 µM Molidustat. After 16 ± 2 hrs cells were harvested and lysed. 30 µg cell extract were separated on a 12% SDS PAGE gel and analyzed by western blot. Samples were probed with a polyclonal antibody directed against phospho-p38 (1:1000). The lower panel shows the blot after stripping and detection of β-actin (1:1000) as a loading control. The panel shows a representative blot of three with similar results. **(B)** The graph shows the relative phosphor-p38 expression calculated by measuring the integrated densities as compared to un-treated samples (defined as 1).

To investigate the molecular mechanism underlying Molidustat-mediated inhibition of TNFα and IL-10, we considered p38 mitogen activated protein (MAP) kinase activation because prolyl-hydroxylase inhibitors were previously implicated in *Escherichia coli*-mediated p38 MAPK activation (27). For this set of experiments, we used a human macrophage-like cell line (THP-1), since GM-CSF required for the maturation of peripheral blood monocytes already activates the MAP kinase pathway precluding studies on *Mtb*-specific regulation (28). THP-1 cells were stimulated with *Mtb*-extract in the presence or absence of Molidustat and cell lysates were analyzed for the expression of phospho-p38. The stabilization of HIF-1α by Molidustat- as shown for primary macrophages- could not be experimentally demonstrated for THP-1 cells due to technical obstacles. *Mtb*-extract induced a profound up-regulation of p38 phosphorylation (**Figure 4**). Molidustat (10 µM) reduced the *Mtb*-mediated up-regulation of phospho-p38 to the levels observed in the untreated control (**Figures 4A, B**).

Taken together these results demonstrate that pharmacological HIF-stabilization inhibits the *Mtb*-induced release of immune-modulatory cytokines and this effect may be associated with an inhibition of the p38 MAP kinase signaling pathway.

### Molidustat Induces the Up-Regulation of the VDR and hBD-2

**Figure 5:**
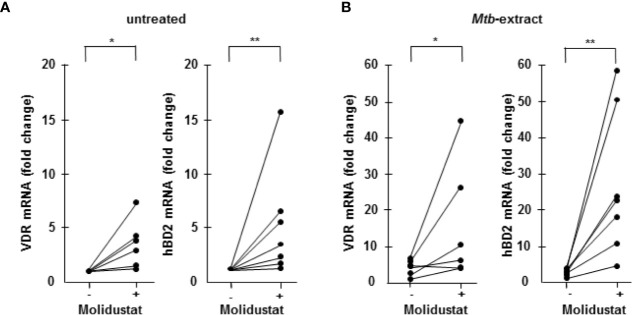
Molidustat induces the up-regulation of the Vitamin D receptor and hBD2 6 x 10^5^ Macrophages were either left untreated or incubated with 10 µM Molidustat for 48 h. Vitamin D receptor (VDR) and human β defensin 2 (hBD2) mRNA levels were measured by LightCycler PCR in **(A)** unstimulated as well as **(B)** *Mtb*-extract- (10 μg/ml) stimulated macrophages. VDR and hBD2 mRNA levels in all probes were compared to levels of unstimulated macrophages without Molidustat treatment, which were defined as 1. The graph shows the mRNA levels for 6 (VDR) or 7 (hBD2) independent donors, respectively. Asterisks indicate statistically significant differences as determined by Wilcoxon test. *p ≤ 0.05; **p ≤ 0.01.

One important effector pathway of human macrophages is initiated by Toll like receptor ligation and involves the up-regulation of the VDR and the subsequent production of the antimicrobial peptide hBD-2 (29). Recently we and others demonstrated that hypoxia similarly up-regulates the VDR and hBD-2 (12, 30, 31). To evaluate whether mimicking hypoxia by pharmacological HIF-1 stabilization has comparable effects, macrophages were incubated with Molidustat and VDR- and hBD2 mRNA levels were determined by quantitative PCR after 48 hrs. In 6 independent experiments Molidustat significantly enhanced VDR (3.5 ± 0.8-fold, *p*=0.02)- and hBD-2 (5.2 ± 1.8-fold; *p*=0.008) expression as compared to un-treated control cultures (**Figure 5A**). To relate this finding to *Mtb*-infection we co-incubated macrophages with *Mtb*-extract and Molidustat. Again, Molidustat strongly enhanced the expression of VDR (4.6 ± 1.8-fold, *p* =0.03) as well as hBD-2 mRNA-expression (11.1 ± 2.9-fold; *p*=0.008) as compared to samples that were treated with *Mtb*-extract alone (**Figure 5B**). Taken together we demonstrated Molidustat results in an increased expression of two molecules (VDR and hBD2) which define an antimicrobial pathway in human macrophages.

### Molidustat Reduces the Growth of Intracellular *Mtb*

**Figure 6:**
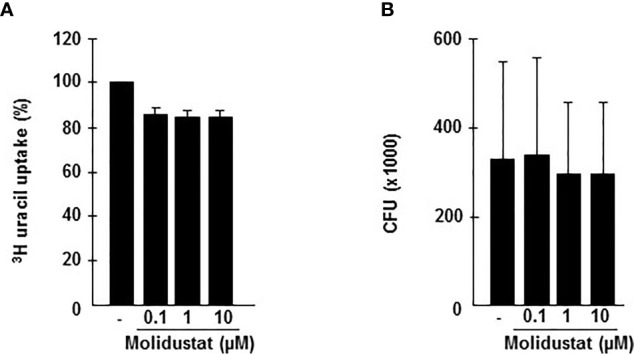
Molidustat does not affect viability of extracellular *Mtb*. **(A)** 1 x 10^5^ *Mtb* H37Rv were grown in 7H9 broth in absence or presence of increasing concentrations Molidustat (0.1, 1, 10 µM) for 5 days at 37°C. Subsequently mycobacteria were harvested and 10 µl of the sonicate were plated on 7H11 agar plates. After 14 d incubation at 37°C CFU were determined. **(B)** The graph shows the results for 5 independent experiments using the identical *Mtb* H37Rv stock.

**Figure 7:**
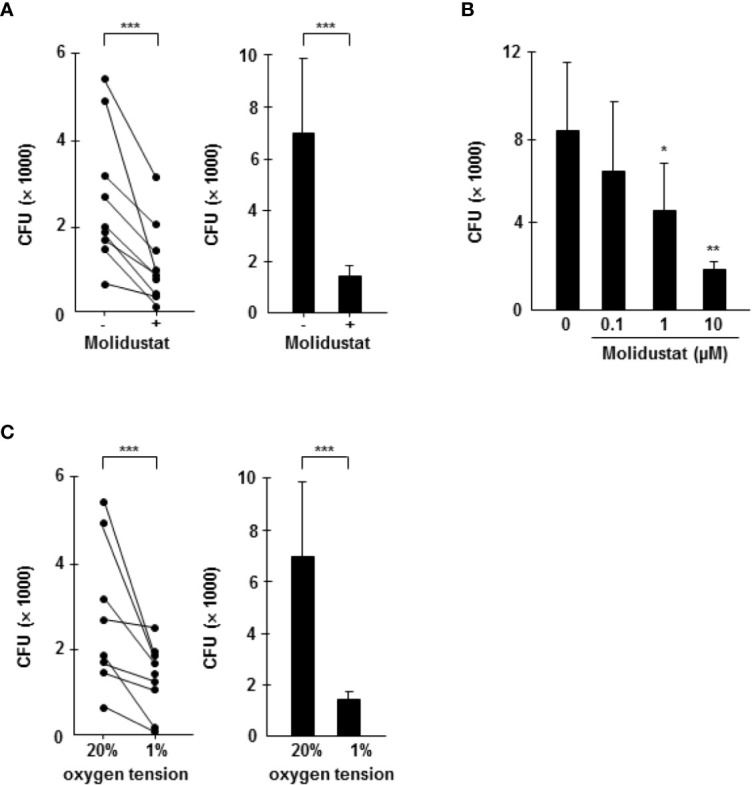
Molidustat mimics hypoxia and inhibits the proliferation of intracellular *Mtb* in a dose dependent manner. Macrophages were infected in bulk culture overnight (MOI 10), harvested, and replated in 24-well plates (2 x 10^5^/300 µl). Infected cells were cultured for 5 days in absence or presence of Molidustat **(A, B)** or under hypoxia **(C)**. The number of viable bacilli was determined after 2 weeks by plating cell lysates on 7H11 agar plates. **(A)** Effect of 10 µM Molidustat on the intracellular growth of viable *Mtb*. *Left panel*: Individual results of 9 out of 11 donors. *Right panel*: Summary of the results of all 11 donors (mean ± SEM). **(B)** Dose dependent effects of 0.1, 1 and 10 µM Molidustat on the intracellular growth of viable *Mtb*. The graph shows the summary of the results of 9 independent donors (mean ± SEM). **(C)** Effect of hypoxia (pO_2_ = 1%) on the intracellular growth of viable *Mtb*. *Left panel*: Individual results of 9 out of 11 donors. *Right panel*: Summary of the results of all 11 donors (mean ± SEM). Asterisks indicate statistically significant differences as determined by Wilcoxon test. *p ≤ 0.05, **p ≤ 0.005, ***p ≤ 0.0005.

The differential effects of Molidustat on cytokine release (inhibition) and the expression of the antimicrobial peptide hBD2 (increase) in macrophages raised the question on the effect on the intracellular growth of *Mtb*. First, we investigated the effect of Molidustat on the proliferation of extracellular *Mtb* in liquid culture. Molidustat (0.1 µM to 10 µM) did not affect the viability of extracellular *Mtb* as measured by comparing the metabolic activity (**Figure 6A**) and growth (colony forming units, **Figure 6B**) in treated and un-treated control cultures. To evaluate the effect on the growth of intracellular *Mtb*, macrophages were infected with virulent *Mtb* and cultured in the presence of Molidustat for 5 days. As observed in un-infected cells (**Figure 1**) Molidustat did not affect the viability of *Mtb-*infected macrophages after 5 d of infection and was in a range between 64-79% (data not shown). The number of viable bacilli was significantly lower in Molidustat- treated macrophages in all 11 donors included in the study (79 ± 5%; *p* = 0.0005) (**Figure 7A**) and this effect was dose dependent (n=9) (**Figure 7B**). The extent of growth inhibition induced by Molidustat closely correlated with the levels of growth inhibition induced by hypoxia (1%) in the same donors (**Figure 7C**). Hypoxia did not influence the viability of macrophages as shown previously (12). By inference this supports our hypothesis that Molidustat enhances macrophage activity against *Mtb* by stabilization of HIF, thereby mimicking an oxygen-restricted environment.

**Figure 8:**
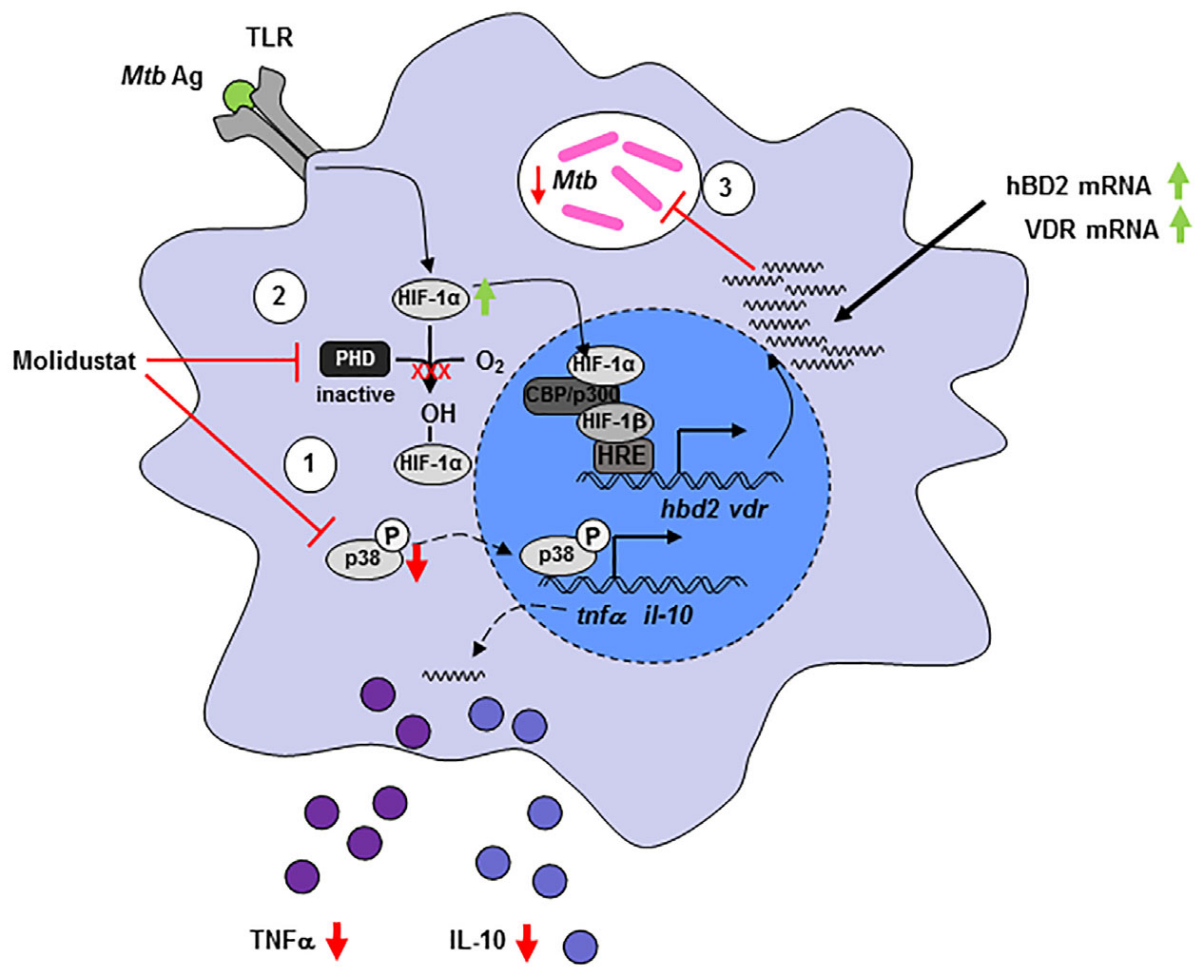
Molidustat directly reduces viability of intracellular *Mtb* and increases the expression of VDR and hBD2, which inhibits growth of *Mtb* in human macrophages. Molidustat interferes in the interplay between *Mtb* and macrophages on several different levels. (1) Application of Molidustat reduces the release of pro- (TNF-α) and anti-inflammatory (IL-10) cytokines released by *Mtb* antigen stimulated macrophages. This effect is a result of interference with the MAPK pathway *via* inhibition of p38 phosphorylation. (2) Inhibition of Prolylhydroxylases by Molidustat prevents degradation of HIF-1α and stabilizes *Mtb* antigen mediated induction of HIF-1α *via* TLR-signaling. (3) HIF-1α is subsequently translocated into the nucleus and induces the expression of an antimicrobial pathway involving VDR and hBD2, which leads to a significant inhibition of intracellular *Mtb* proliferation.

In summary, our experiments demonstrate that pharmacological HIF-stabilization by Molidustat has profound effects on macrophage functions (summarized in **Figure 8**). The skewing of the expression of cytokines and antimicrobial peptides ultimately results in reduced growth of intracellular *Mtb*. These findings highlight the potential of HIF-stabilizers to serve – in addition to the established benefit for ameliorating renal anemia – as host-targeted therapy to treat tuberculosis when classical antibiotic regimes fail as a result of antibiotic resistance.

## Discussion

Treatment of tuberculosis remains a major challenge due to the emergence of drug-resistance strains, severe side effects of first-line drugs and lack of compliance related to the 6-months duration of treatment. Host-directed therapies that support the immune system rather than directly acting on the pathogen provide an attractive alternative treatment strategy. Here, we demonstrate that pharmacological stabilization of hypoxia-induced factor by Molidustat, a compound already tested in phase III clinical trials for the treatment of renal anemia, modulates *Mtb*-associated macrophage functions resulting in reduced growth of the pathogen. Our results provide preclinical evidence that the hypoxia-triggered signal transduction pathway is a potential target for host-directed therapies against intracellular bacteria, specifically *Mtb*.

HIF-1α is an essential environmental and metabolic sensor that acts as a transcription factor thereby affecting multiple immune cell functions. Physiologically HIF-1α is stabilized at low oxygen tension (14, 32). However, HIF-1α stabilization may also occur at physiological oxygen levels, when target cells are stimulated, for example by *Mtb*-derived trehalose dimycolate (33) or TLR4 ligation (16). Stabilization is linked to the transcription factor NF-κB, which activates the downstream target HIF-1α (34). Accordingly, the human HIF-1α promotor contains a canonical NF-κB binding site 197/188 bp upstream of the transcription start site (35). Here, we demonstrate that the prolyl hydroxylase inhibitor Molidustat increases HIF-1α levels in human macrophages stimulated with mycobacterial antigens. Molidustat also upregulates HIF-2α in epithelial (HeLa)-, adenonocarcinoma (A549)- and hepatoma (Hep3B) cell lines. Since Molidustat stabilizes both HIF isotypes, the effects observed in this study cannot definitively be attributed to HIF-1α. However, HIF-2α is most abundantly expressed in vascular endothelial cells, plays an important role in embryogenesis and is closely related to VEGF mRNA expression (36, 37). Therefore, it is likely that the effects of Molidustat on macrophages are mediated by HIF-1α.

HIF-1α activation switches energy metabolism to glycolysis to provide continuous ATP supply, when oxygen is limited (38). As compared to lymphoid cell lines, which predominantly use oxidative phosphorylation for their energy metabolism and therefore rely on the presence of high levels of oxygen, myeloid cells (neutrophils, macrophages) favor glycolysis (39). This is in accordance with our observation that macrophages tolerate high concentrations of Molidustat (10µM) (**Figure 1**), resulting in a “hypoxia-like” microenvironment.

In response to microbial stimuli macrophages release a broad panel of inflammatory mediators. In tuberculosis macrophage-derived TNFα and IL-10 play a special role and the balance between these mediators affect the outcome of tuberculosis infection. p38 – a key kinase of the MAP-kinase pathway – regulates TNFα and IL-10 release (40). In addition, restriction of local oxygen supply increases the production of cytokines and chemokines (41, 42). In our study Molidustat decreased both *Mtb*-induced TNFα and IL-10 secretion by macrophages (**Figure 3**). Similarly, AKB-4924, another pharmacological HIF-1α stabilizing compound, dampened the host inflammatory response (27). Intriguingly this effect was also attributed to the inhibition of p38 MAPK activation (27). Hence inhibition of p38 phosphorylation seems to be a common effect of pharmacological HIF-stabilizers.

At first glance the differential regulation of immune molecules appears contradictory. However, HIF-1-α is a ubiquitous transcription factor which is expressed in all myeloid cells including macrophages. The genes responding to HIF-1-α are highly diverse and include the regulation of metabolism, cell division and immune functions (6). The functional impact of gene regulation does not follow a predictable pattern and the understanding of biological end points (e.g. cytokine release or expression of antimicrobial peptides) requires specific experimental approaches. We believe that the differential effects on cytokine release and VDR/hBD2 reflects the complexity of biological effects governed by HIF-1-α.

Our study was not designed to define the molecular mechanism of Molidustat/HIF-1α-mediated growth inhibition of *Mtb*. We hypothesize that HIF-1α-mediated modifications of the energy metabolism in macrophages interfere with the multiplication of *Mtb*. First, increased intracellular HIF-1α levels promote glycolysis resembling the Warburg effect in tumors (43). Increased glucose consumption results in increased formation of lactate (44). Several reports indicate that lactic acid monomers activate macrophages and enhance their ability to kill intracellular *Mtb* (45). Second, increased levels of HIF-1α induce a metabolic reprogramming of the citric acid cycle that affects the production of critical metabolites such as succinate (46) and itaconic acid (47). Succinate serves as an inflammatory signal which also stabilizes HIF-1α (48). Itaconic acid, which is formed *via* decarboxylation from the tricarboxylic acid cycle intermediate cis-aconitate directly inhibits growth of *Mtb.* Mechanistically this effect is mediated by inhibition of the bacterial isocitrate lyase, a key enzyme of the glyoxylate shunt responsible for bacterial growth (47). In mice HIF-1α upregulation and glucose metabolism were shown to be essential for macrophage migratory activity (7, 49). Alternative mechanisms for HIF-1α mediated antimicrobial activity could be the induction of antimicrobial peptides (CRAMP), granule proteases (Cathepsin G) as well as iNOS producing nitric oxide in mice. These mechanisms contributed to HIF-1α- dependent bactericidal activity and systemic spread of group A streptococci, *Salmonella Typhimurium* and *Pseudomonas aeruginosa* (15). Similarly, indirect (Molidustat)- or direct exposure to hypoxia (hypoxia chamber) triggered the expression of VDR and its downstream target hBD2 thereby enhancing the antimicrobial activity against intracellular *Mtb* (**Figure 5**) (12). Notably hBD2 has been directly associated with growth restriction of intracellular *Mtb* (50).

Although Molidustat did not affect the growth of *Mtb* (**Figure 6**), direct effects of HIF-1 stabilizers on *Mtb* cannot be excluded. Even though the precise mode of action for inhibiting Prolylhydroxylase II by HIF-1α stabilizers is unknown, two major principles are currently discussed: Blocking the active site of the enzyme (e.g. FG-4497) or chelate Fe^2+^ (e.g. Deferoxamine), which is essential for the activity of prolyl hydroxylase. In case of L-Mimosine, a Molidustat-related HIF-1α stabilizer, both mechanisms are active (51, 52). Intriguingly iron chelating agents such as deferoxamine significantly decrease the viability of extracellular *Mtb* (53). Since Molidustat, as compared to deferoxamine, is able to permeate cellular membranes, direct effects on intracellular *Mtb* mediated by partial Fe^2+^-chelation may be possible.

Our results support an emerging concept of a role for HIF-1α in protection against bacterial infections. Other studies demonstrated that HIF-1α supports the clearance of bacterial infections in mouse keratinocytes infected with Group A streptococci, a *Mycobacterium marinum* zebrafish model or infection of murine bladders with uropathogenic *Escherichia coli* (17, 27, 54). Accordingly, the HIF-1α stabilizer (AKB-4924) supported the clearance of *Escherichia coli* (27). Even though our results do not provide direct proof for a causative link between reduced cytokine release and p38 MAP kinase expression, we suggest that the “hypoxia-HIF-1α-p38 Map kinase axis” is a novel and intriguing target for host directed therapy of bacterial infections. Appropriate compounds, such as Molidustat used in this study, are well tolerated *in vivo*, currently investigated in Phase II/III clinical trials (20, 55) or – such as Roxadustat – already prescribed (22) to treat renal anemia. Our study should therefore encourage clinical studies to extend the clinical application of HIF-1α stabilizers for the treatment of severe bacterial infections.

## Data Availability Statement

The raw data supporting the conclusions of this article will be made available by the authors, without undue reservation.

## Ethics Statement

PBMC were isolated from buffy coat preparations from anonymous donors (Institute of Transfusion Medicine, Ulm University) which cannot be tracked. The ethics committee of the University Hospital in Ulm declared that no formal ethical approval is required for this study.

## Author Contributions

SZ: planned and conducted experiments, wrote the manuscript. SH: conducted experiments. DM: conducted experiments. MG: conducted experiments. SS: concepted and supervised the study, wrote the manuscript. All authors contributed to the article and approved the submitted version.

## Funding

This work was supported by the German Research Foundation (STE 925/4-1 and CRC1279) and the Landesstiftung Baden-Württemberg (Förderprogramm Biotechnologie).

## Conflict of Interest

The authors declare that the research was conducted in the absence of any commercial or financial relationships that could be construed as a potential conflict of interest.

## Abbreviations

CFU, colony forming units; hBD2, human ß defensin 2; HIF, Hypoxia inducible factor; MOI, multiplicity of infection; *Mtb*, *Mycobacterium tuberculosis*; PBMC, peripheral blood mononuclear cells; VDR, Vitamin D receptor.
